# Microbiota diversity and differences in the respiratory tract of children with pneumonia

**DOI:** 10.1002/ped4.12466

**Published:** 2025-02-14

**Authors:** Dandan Ge, Lingyun Hou, Jintao Guo, Xuejing Lv, Yungang Yang

**Affiliations:** ^1^ Department of Pediatrics, Pediatric Key Laboratory of Xiamen, The First Affiliated Hospital of Xiamen University, School of Medicine Xiamen University Xiamen Fujian China; ^2^ Institute of Pediatrics, School of Medicine Xiamen University Xiamen Fujian China; ^3^ Department of Pediatrics Linyi People's Hospital Linyi Shandong China; ^4^ National Institute for Data Science in Health and Medicine School of Medicine Xiamen University Xiamen Fujian China

**Keywords:** Lower respiratory tract, Microbiota, Pneumonia, Upper respiratory tract

## Abstract

**Importance:**

Although studies have examined the link between microbiota and airways, the understanding of microbial imbalances in the upper respiratory tract (URT) and lower respiratory tract (LRT) in pediatric pneumonia remains limited.

**Objective:**

To elucidate the microbial communities within these areas, shedding light on the microbiota's contribution to pneumonia progression and the underlying metabolic shifts.

**Methods:**

Pharyngeal swabs and bronchoalveolar lavage fluid samples were gathered from children with pneumonia and sequenced for 16S rDNA gene. Microbiota composition and differences between URT and LRT were analyzed.

**Results:**

*Proteobacteria* (40.91%), *Firmicutes* (25.61%), and *Actinobacteria* (12.77%) were the three most abundant phyla in the airways of the children with pneumonia. Richness (*P *= 0.003), Chao1 (*P *= 0.003), and abundance‐based coverage estimator (*P *= 0.003) indices were significantly higher in the LRT than URT. *Streptococus* *infantis* was more abundant in the URT, whereas *Cyanobacteria* at the phylum level, *Alphaproteobacteria* and *Chloroplast* at the class level, *Pseudomonadales*, *Burkholderiales*, and *Streptophyta* at the order level, *Moraxellaceae* and *Corynebacteriaceae* at the family level, *Moraxella* and *Corynebacterium* at the genus level were more prevalent in the LRT. Multiple pathways such as d‐glutamine and d‐glutamate metabolism (*P *= 0.0032) were significantly activated in the URT, whereas inorganic ion transport metabolism (*P *= 0.0239) and tryptophan metabolism (*P *= 0.0284) were significantly activated in the LRT. *Streptococcus* genus negatively impacted blood indicators in those children.

**Interpretation:**

Our study characterizes the LRT and URT microbiota in pediatric pneumonia children and links them to clinical features, enhancing our understanding of the disease's pathogenesis.

## Graphical abstract

Differences in microbial communities in the upper and lower respiratory tract in patients with pneumonia were investigated in this study by sequencing the 16S rRNA genes in pharyngeal swabs and alveolar lavage fluid. Forty‐three subjects were enrolled in the study, and one upper and one lower respiratory tract sample were collected from each subject, resulting in a total of 86 samples. A total of 86 samples were collected in the study, excluding 24 samples with failed RNA quality control (QC), 62 samples were sequenced on the machine, and after sequencing, 6 samples were excluded from sequencing quality QC, and finally, 56 samples were included in the group for analysis. A total of 56 samples consisted of 28 from alveolar lavage fluid (LRT) and 28 from pharyngeal swabs (URT) of 28 children with pneumonia.

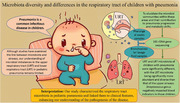

## INTRODUCTION

Pneumonia is a lung inflammation triggered by various pathogens or other contributing factors.^1^ Due to the special airway anatomy, physiological and immune characteristics, children's peripheral lung tissue, or bronchi are more vulnerable to pathogenic bacteria than those of adults, making pneumonia a leading cause of illness and death among children golbally.^2^, ^3^ Pneumonia in children is caused by a variety of complex pathogens, including bacteria, viruses, *mycoplasma*, and chlamydia.^4^, ^5^ Clinical manifestations of pneumonia are not solely due to a single bacterial species but reflect a broader shift in the microbiota's composition. Specific regions within the respiratory tract host contain specialized bacterial communities playing a major role in human health maintenance.^6^ Widespread diversity of bacterial communities is essential for maintaining immune homeostasis. Imbalances in the respiratory microbiota have been associated with the development of various diseases, including asthma, pneumonia, and allergic diseases.^7^

Clinical diagnosis often relies on pathogenic diagnosis, yet traditional culture methods fail to capture the full microbial profile in the airways due to the technical challenges in obtaining lower respiratory tract (LRT) samples from children. The application of 16S rRNA gene sequencing has provided a more comprehensive view of airway microbiota composition and distribution. In healthy individuals, the bacterial abundance is higher in the upper respiratory tract (URT) than LRT,^8^ with the respiratory microbiota in the LRT largely derived from the URT, meaning that no species differences exist between the URT and LRT microbiota in healthy individuals. However, ecological dysregulation of the oropharyngeal community of the URT in adults and the elderly can lead to overgrowth of pathogenic bacterial groups and subsequent lung invasion, triggering bacterial pneumonia.^9^ The taxonomic groups of *Haemophilus* and *Pasteurellaceae* have been positively correlated with pneumonia severity in induced sputum specimens from infants and children.^10^ Distinctions in microbiota composition and structure in the LRT of adult patients with pneumonia have been associated with local host immune responses, peripheral metabolic reprogramming, and mortality.^11^ Prevention and therapeutic strategies would aim to reinforce the microbiome and mucosal immunity.^12^ Therefore, studying the composition of the microbiota in children with pneumonia could inform the development of targeted immunotherapies. In this study, we conducted 16S rRNA gene sequencing on URT and LRT samples from children with pneumonia to elucidate respiratory microbiota changes, understand pneumonia pathogenesis, and guide the development of effective clinical interventions.

## METHODS

### Ethical approval

The study was approved by the Medical Ethics Committee of the First Hospital of Xiamen University ([2023] Research Ethics Review No. (094)). Affirmative informed consent was obtained from the patients or guardians for the study. All the procedures performed in this study complied with the ethical standards of the committee.

### Study subjects

The patients who were hospitalized for pneumonia and underwent bronchoscopy in the Department of Pediatrics of the First Affiliated Hospital of Xiamen University from July 2016 to July 2018 were included. A diagnosis of pneumonia in children was confirmed based on the presence of the following symptoms: (1) fever, cough, and wheezing. Older children may experience chest pain, but hemoptysis is rare. Infants younger than 2 months may have no fever and may exhibit spitting, breath‐holding (apnea), or choking. Persistent fever with cough for >3–5 days. (2) Rapid respiration and wet rales, rapid respiratory rate standard: 1‐min observation in calm: less than 2 months old ≥60 times/min; 2 months old to 1 year old ≥50 times/min; 1 year to 5 years old ≥40 times/min; and 5 years old and above ≥30 times/min. (3) Typical chest imaging changes (interstitial infiltrates, segmental and lobar consolidation, and pulmonary lymph node enlargement). Exclusion criteria include incomplete medical records; antibiotics used before taking the sample; duplicate collection within 24 h; poor sample quality; unmatched samples that bronchoalveolar lavage fluid (BALF) only or throat swab only, and those that are unlabeled or mislabeled.

### Sample collection and DNA extraction

We collected newly admitted patients diagnosed with pneumonia, and patients undergoing bronchoscopy were required to fulfill a 4–6 h water fasting. Those who met the requirement on the day of admission proceeded directly to bronchoscopy, whereas those who did not scheduled for the following day. All patients had a pharyngeal swab taken before the commencement of bronchoscopy.

Pharyngeal swab collection method: check the patient's information, instruct the child to open his mouth and make the “ah” sound, wipe the secretion with a long sterile swab in the child's pharynx, and place the swab into the culture tube after gently wiping. After collection, the samples were transferred to −80°C freezer within 30 min.

Bronchoscopy operation method: children fasting water for 4–6 h before surgery, atropine subcutaneous injection 0.01–0.02 mg/kg (maximum dose 0.5 mg) to reduce airway secretion, 2% lidocaine 2 mL nebulization, midazolam 0.1–0.3 mg/kg intravenous injection, the use of domestic vision of the new QG3320/QG3430 (Zhuhai Vision Medical Technology Company, Zhuhai), according to the age of the selection of the appropriate type of bronchoscope. The patient takes the supine position, inserts the bronchoscope through the nose, sprays 2% lidocaine surface anesthesia locally while entering the scope, observes the vocal folds, trachea, rumpus, and each lobe bronchus in turn, and carries out the bronchoalveolar lavage and other inspections and treatments as needed. For patient who performed intubation after admission, bronchoscopy was done with the introduction of an endotracheal tube instead of nasal. Bronchoscopy was carried out in an independent operation room.

After the pharyngeal swabs were dissolved in the solution, they were centrifuged at 14 000 rcf for 20 min at 4°C to retain the cellular precipitate. Total genomic DNA was extracted from pharyngeal swabs and BALF using the Qiagen‐purified pathogen DNA kit, following the manufacturer's instructions.

### 16S ribosomal DNA gene sequencing and quality control

Upon completion of DNA extraction, the V3–V4 region of 16S rDNA was targeted for amplification using the universal primers 341F and 806R.^13^ PCR amplification was performed using a diluted DNA template and high‐fidelity enzyme. A total of 86 samples were amplified, of which 24 did not meet the quality criteria and were excluded, resulting in 62 samples that were ultimately sequenced. Water without DNA template was used as a negative control when sequencing the 16S rDNA gene to detect potential contamination during the experiment. The raw data was uploaded in NCBI (accession number PRJNA982313).

### Sequence data processing and analysis methods

The quality of the data was checked using sample rarefaction curves (Figure S1). Species accumulation plots show the increase in operational taxonomic units (OTUs) detected with the addition of each sample (Figure S2). Once the raw sequencing data were fused and chimeras removed, all sequences were homologated and clustered into OTUs based on 97% sequence similarity threshold using the QIIME2 cluster method, with each OTU representing a distinct bacterium type. The data were then visually analyzed in conjunction with R, Tax4Fun2, Phyloseq, and DESeq2.

Species abundance and diversity in microbial communities were assessed using alpha diversity analysis, mainly from the Richness, Chao1, and abundance‐based coverage estimator (ACE) indices, which were positively correlated with the diversity of species in the community; higher values of these indices indicate a richer species composition within the community. Shannon, Simpson, and InvSimpson indices were used to evaluate microbial community diversity within the samples. Higher Shannon indices indicate higher community diversity. Simpson and InvSimpson indices were negatively correlated with community diversity, with higher indices indicating lower community diversity.

Microbial community differences were analyzed using the Bate diversity assessment. Unweighted UniFrac and Bray–Curtis analyses were used to compare the presence or absence of significant microbial community differences between samples in specific evolutionary lineage species by calculating the evolutionary information between each sample sequence. The unweighted UniFrac or Bray–Curtis distance matrix was obtained by a comparative analysis between the samples, and the results were visualized and presented by principal coordinate analysis (PCoA).

Linear discriminant analysis (LDA) was employed to quantify the impact of component richness on sample differentiation and to pinpoint communities or species that exert a substantial influence on the delineation of samples.

The metabolic function of the bacterial population was predicted using the Tax4Fun2 package, where the 16S rDNA sequences were first compared to a built‐in reference sequence to obtain species annotations based on sequence similarity. The communities’ functional profile was then determined by integrating the functional characteristics of the species genomes with species abundance data from the OTU table. The relative pathway abundances for each sample were obtained by mapping the functional gene abundances to their corresponding metabolic pathways, specifically KEGG pathway at the KO level 3 taxonomic unit (Table S1). Following the acquisition of community functional abundance tables, intergroup disparities were analyzed using STAMP software. Finally, the differences in functional gene abundance across the enriched pathways were interpreted according to the KEGG database pathways.

### Statistical analysis

Data were analyzed using R (version 4.2.0). Continuous variables are shown as standard mean ± standard deviation, and the Kruskal–Wallis or Mann–Whitney tests were used to assess group differences. Categorical variables are shown as counts and percentages or medians and interquartile ranges, and the chi‐square test or Fisher's exact test was used to test for differences between groups. The differences between between‐ and within‐group variations in beta diversity were examined using the Adonis test. PCoA was used to visualize the results of the analyses. Species differences between groups were analyzed using the LDA effect size (LEfSe). This analysis initially used a nonparametric Kruskal–Wallis rank sum to detect species with significant differences in abundance disparities among subgroups and then used a Wilcoxon rank sum to test the consistency of the differences in the different species from the previous step in the different subgroups of the intergroups. Finally, a linear regression analysis was utilized to estimate the impact of species abundance on the differential effects of each component. Intergroup metabolic pathway analyses were performed using the STAMP software. All statistical tests were conducted as two‐sided, and statistical significance was set at *P* < 0.05.

## RESULTS

### Population and clinical characteristics

**FIGURE 1:**
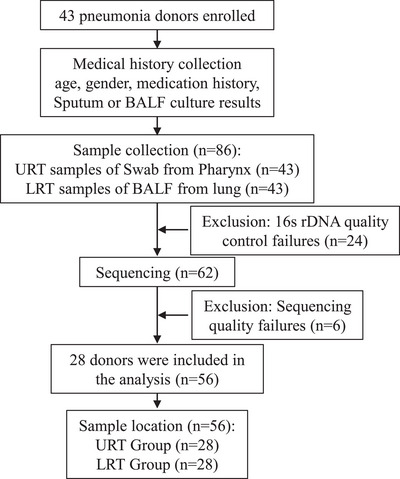
Flow diagram of the pneumonia recruitment and grouping process. BALF, bronchoalveolar lavage fluid; LRT, lower respiratory tract; URT, upper respiratory tract.

Differences in microbial communities between the URT and LRT in patients with pneumonia were investigated in this study by sequencing the 16S rDNA genes from pharyngeal swabs and BALF. Forty‐three patients were enrolled, and one URT and one LRT sample were collected from each patient, resulting in a total of 86 samples. After excluding 24 samples due to failed DNA quality control (QC), 62 samples were processed for sequencing. Following sequencing, an additional 6 samples were excluded based on sequencing QC, leaving 56 samples for analysis. These 56 samples consisted of 28 from alveolar lavage fluid (LRT) and 28 from pharyngeal swabs (URT) of 28 children with pneumonia. The pediatric pneumonia cohort comprised 16 males and 12 females, resulting in a male‐to‐female ratio of approximately 1.33:1. The age distribution of the children ranged from 32 days to 11 years, with a mean age of 2.3 years and a median age of 7.5 months. The pneumonia classification was determined based on the sputum culture, nucleic acid, or antibody testing. The process of sample inclusion for pneumonia is illustrated in Figure 1. Patient characteristics and outcomes are shown in Table S2.

### Microbial communities’ abundance and composition in the URT and LRT of patients with pneumonia were significantly different

**FIGURE 2:**
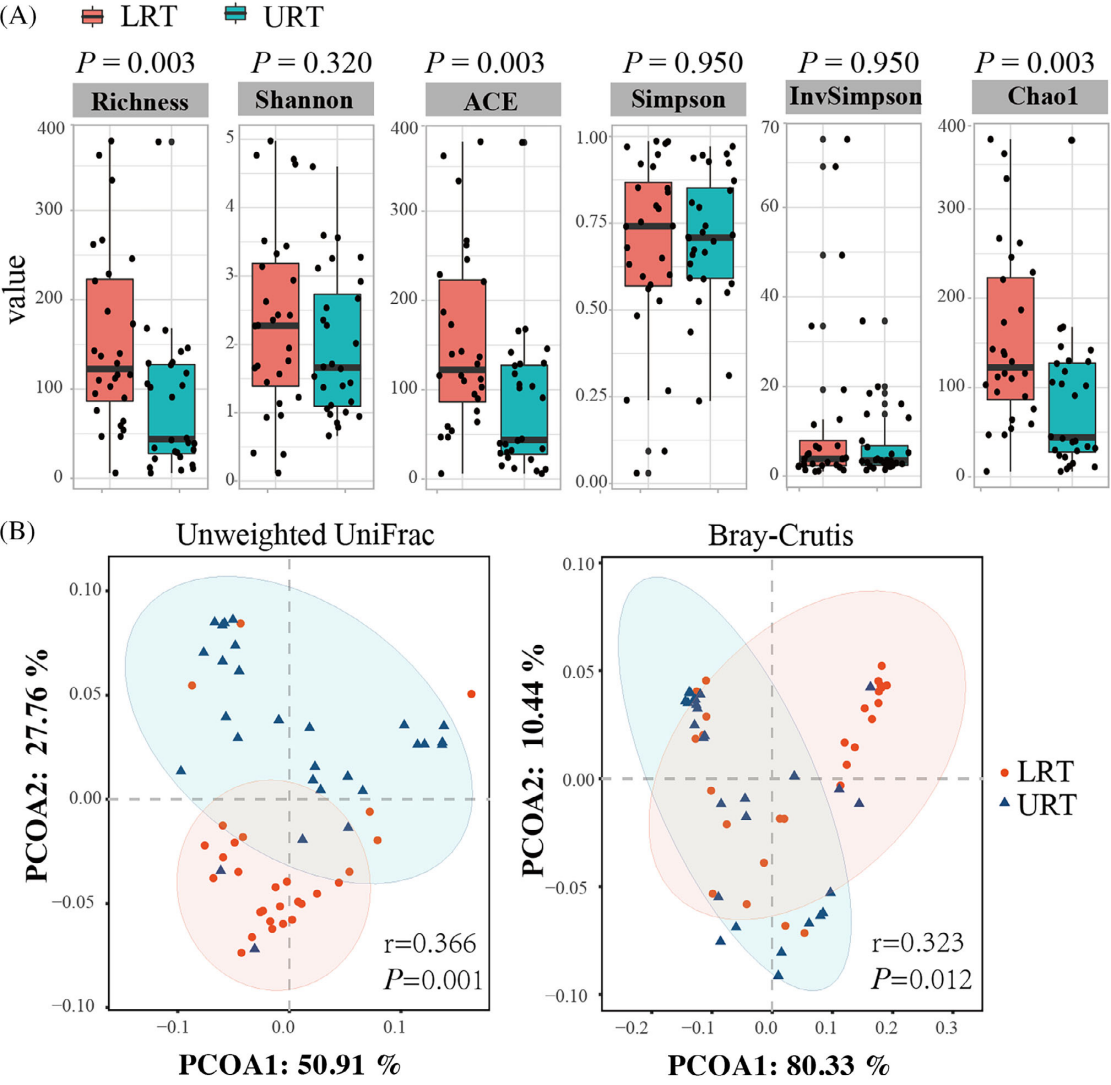
Overall differences in the airway microbiota of patients with pneumonia. (A) Differences in the abundance and diversity of microbial communities based on the alpha diversity index. (B) PCoA analysis of microbial community composition and abundance between the LRT and URT groups. Left, unweighted UniFrac; right, Bray–Curtis. The horizontal and vertical coordinates in the PCoA plot represent principal components contribution, expressed as a percentage; each point on the plot represents a sample, with distances positively correlated with bacterial community differences. LRT, lower respiratory tract; PCoA, principal coordinate analysis; URT, upper respiratory tract.

The sequenced samples were filtered to obtain 6 424 701 high‐quality sequences, with an average of 114 727 sequences per sample. Using rDNA sequence similarity greater than 97% as the criterion for defining OTUs, a final total of 5544 OTUs were identified, with an average of 99 OUTs per sample. Alpha diversity indices were used to calculate microbial richness and diversity. Species richness indices, including Richness (*P *= 0.003), ACE (*P *= 0.003), and Chao1 (*P *= 0.003), were significantly higher in the LRT than in the URT group (Figure 2A).

PCoA of beta diversity and analysis of similarity (Adonis) tests showed significant differences in microbial composition and abundance between the LRT and URT groups (unweighted UniFrac, *P *= 0.001; Bray–Curtis, *P *= 0.012) (Figure 2B).

### Microbiota composition in the URT and LRT of patients with pneumonia

**FIGURE 3:**
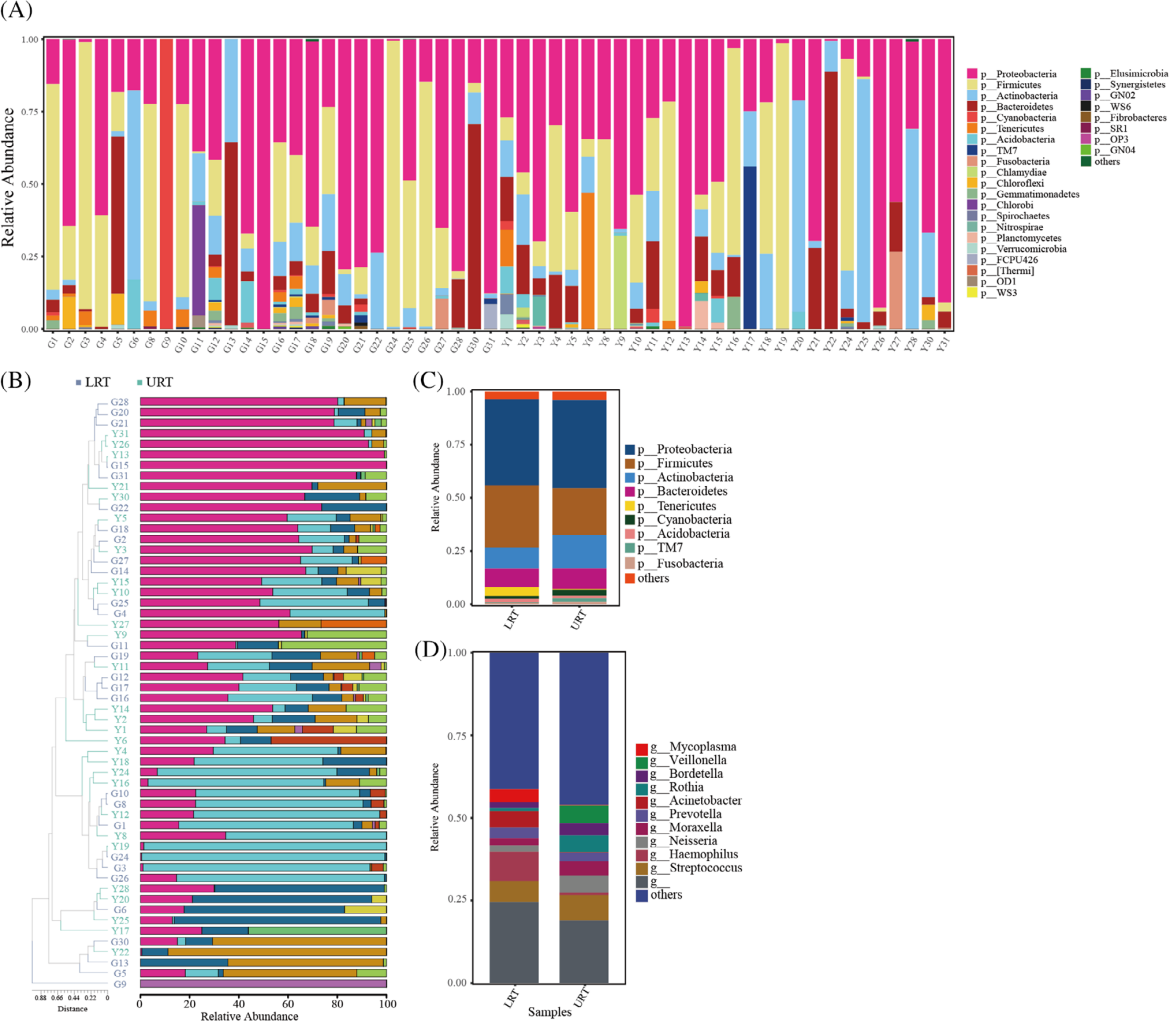
Analysis of the airway community in patients with pneumonia. (A) Composition and abundance of the respiratory tract microbiota of patients with pneumonia (*Note*: horizontal coordinates are phyla; vertical coordinates are abundance); (B) Clustering structure and abundance profiling of all the samples; (C) Relative distribution of the most abundant phyla in the URT and LRT. (D) Proportion of genus with the highest abundance in the URT and LRT. LRT, lower respiratory tract; URT, upper respiratory tract.

Based on the phylogenetic positioning of the 358 OTUs in the evolutionary tree of the respiratory tracts of patients with pneumonia, a total of 33 phyla were identified across the bacterial communities, with 21 phyla present in the URT and 31 phyla in the LRT. The relative abundance of each phylum in the samples is shown in Figure 3A. The nine most abundant phyla were *Proteobacteria, Firmicutes, Actinobacteria, Bacteroidetes, Tenericutes, Cyanobacteria, Acidobacteria*, *TM7*, and *Fusobacteria* (Figure 3C). Clustering analysis of samples demonstrating the similarity between individual URT and LRT samples (Figure 3B). The phylum *Proteobacteria* was similar in the URT and LRT groups; however, *Firmicutes* was dominant in the LRT group, and *Firmicutes* and *Actinobacteria* was dominant in the URT group (Figure S3). The proportions of genera with the highest abundance in different locations of pneumonia patients are shown in Figure 3D that displays the 10 most abundant genera: *Mycoplasma, Veillonella, Bordetella, Rothia, Acinetobacter, Prevotella, Moraxella, Neisseria, Haemophilus*, and *Streptococcus*.

### Differential bacteria between URT and LRT in children with pneumonia

**FIGURE 4:**
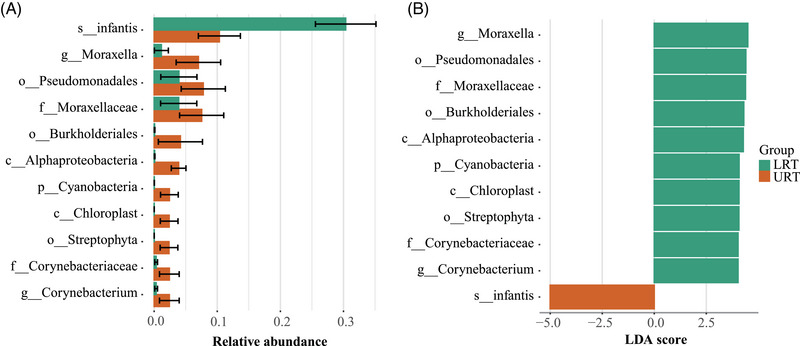
Relative abundance combined with linear discriminant analysis (LDA). (A) Significantly different top 11 taxons in the LRT and URT groups. (B) Bar graph of LDA scores, where the LDA score indicates the effective size and ranking of each differential abundance taxonomic unit (LDA > 4; only absolute values of LDA > 4 are shown in the graph), the color of the bar graph represents the respective group, and the length represents the LDA score, that is, the degree of influence of each taxon that differed significantly between groups. The name of the taxon level is abbreviated as p, phylum; c, class; o, order; f, family; g, genus; and s, species. LRT, lower respiratory tract; URT, upper respiratory tract.

Further analysis revealed significant differences in species composition between URT and LRT of pneumonia patients (*P* < 0.01) (Figure 4 and Table S3). Figure 4 highlights the first 11 taxons that with the most pronounced differences. As shown in Table S3, 21 families, including *Moraxellaceae, Corynebacteriaceae*, and *Rhodobacteraceae*, were significantly different between the URT and LRT. Similarly, 16 genera, including *Moraxella*, *Corynebacterium*, and *Propionibacterium*, were found to be significantly different between the URT and LRT. Seven species, *Streptococcus* *infantis*, *Propionibacterium acnes*, *Bacteroides ovatus*, *Vibrio harveyi, Mycoplasma haemominutum, Brevundimonas vesicularis*, and *Sphingomonas yabuuchiae* were significantly different between the URT and LRT (Table S3). Notably, *Streptococcus infantis* was the only dominant organism found to be markedly higher in the URT than in the LRT. Although, in the LRT, there are many bacteria with significantly higher abundance than in the URT, including *Cyanobacteria* at the phylum level, *Alphaproteobacteria* and *Chloroplast* at the class level, *Pseudomonadales*, *Burkholderiales*, and *Streptophyta* at the order level, *Moraxellaceae* and *Corynebacteriaceae* at the family level, and *Moraxella* and *Corynebacterium* at the genus level (Table S3).

### Remarkable metabolic differences in the URT and LRT in children with pneumonia

**FIGURE 5:**
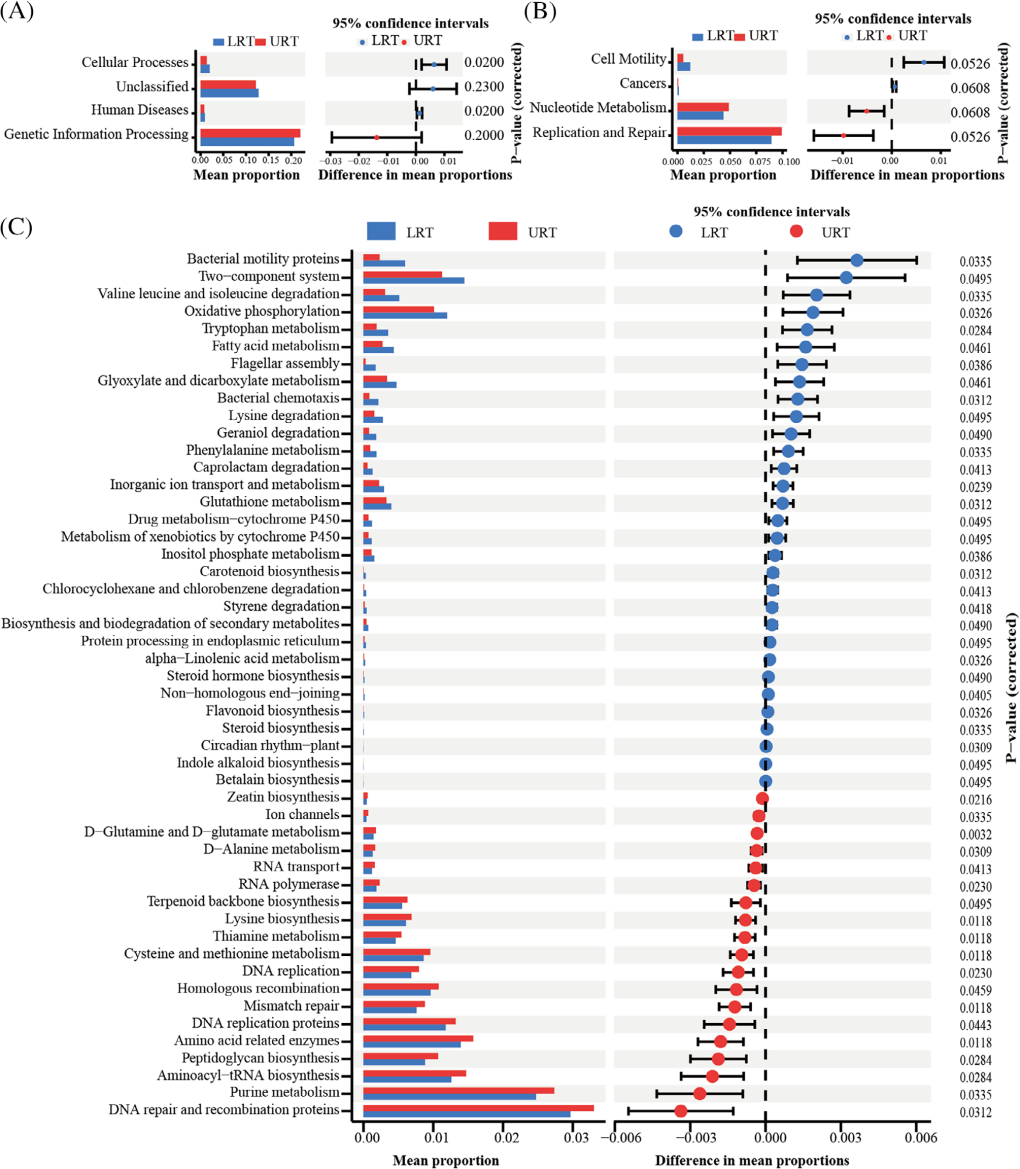
Diagram of the analysis of differences in KEGG metabolic pathways between upper respiratory tract (URT) and lower respiratory tract (LRT). (A) Metabolic pathways and proportions were significantly different at the KEGG L1 level. (B) Metabolic pathways and proportions were significantly different at the KEGG L2 level. (C) Metabolic pathways and proportions were significantly different at the KEGG L3 level (left bar graph shows the presence of differential metabolic pathways and proportions. Blue/red dots on the right indicate metabolic pathways predominant in the UTR or LRT, with a confidence interval of 95%, corrected *P* < 0.05).

Disturbances in respiratory microbiota can lead to shifts of bacterial metabolite concentrations, which in turn can affect the development of disease. Many bacterial metabolites play a role in modulating host metabolic processes and signaling pathways. To investigate the impact of changes in the respiratory microbiota in children with pneumonia, we performed a functional prediction analysis of the metabolic pathways of the microbiota. The metabolic pathways associated with URT and LRT are shown in Figure 5, which illustrates the differences in metabolic pathways at the three hierarchical levels. URT and LRT significantly differed in metabolic pathways at the L1 level with respect to cellular processes and human diseases. No significantly different pathways were observed at the L2 level. Fifty metabolic pathways were identified significantly differentially expression at the L3 level. These included pathways such as d‐glutamine and d‐glutamate metabolism, lysine biosynthesis, and thiamine metabolism. Thirty‐one pathways were significantly enriched in LRT (*P* < 0.05), including bacterial motility proteins, two‐component system, valine leucine, and isoleucine degradation. Nineteen pathways were significantly enriched in URT (*P* < 0.05), including RNA repair and recombination proteins, purine metabolism, aminoacyl‐tRNA biosynthesis, and others. These findings demonstrate that the URT microbiota may activate the d‐glutamine and d‐glutamate metabolism (*P *= 0.0032), lysine biosynthesis (*P *= 0.0118), and thiamine metabolism (*P *= 0.0118) metabolic pathways. The LRT microbiota may activate the inorganic ion transport and metabolism (*P *= 0.0239), tryptophan metabolism (*P *= 0.0284), and circadian rhythm‐plant (*P *= 0.0309) pathways.

### Correlation analysis between microbiota and clinical characteristics

**FIGURE 6:**
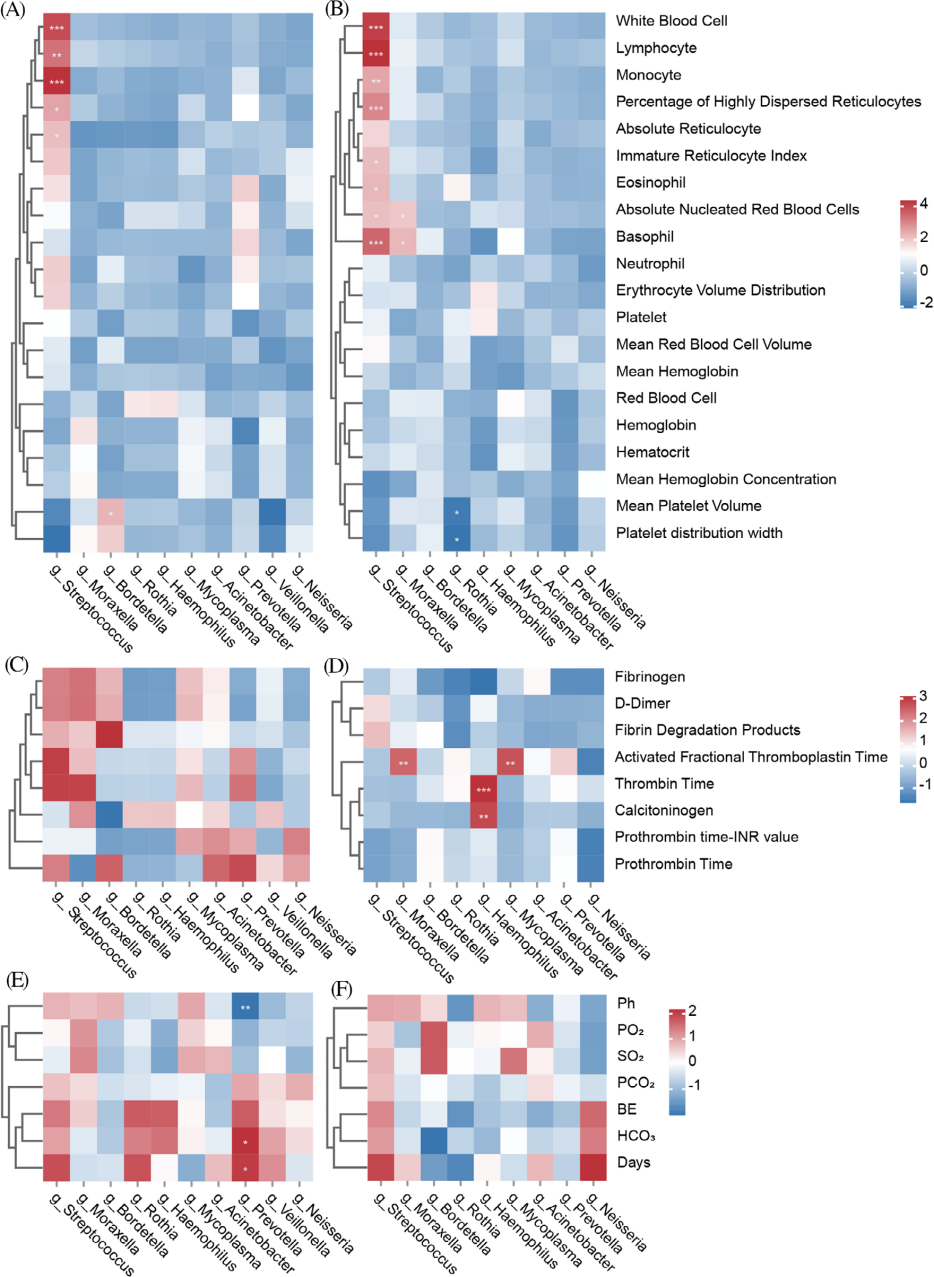
Correlation networks between microbiota and clinical characteristics. (A, C, and E) Correlation between bacterial genera and clinical indicators in the URT. (B, D, and F) Correlation between bacterial genera and clinical indicators in the LRT. PO_2_, partial pressure of arterial oxygen; SO_2_, arterial oxygen saturation; PCO_2_, partial pressure of arterial carbon dioxide; BE, base excess; HCO_3_, carbonic acid; days, hospitalization days (*P < 0.05, **P < 0.01, ***P < 0.001, and ****P < 0.0001). LRT, lower respiratory tract; URT, upper respiratory tract.

To observe the effect of microbiota on clinical characteristics (Tables S4 and S5), we analyzed the correlation between the abundance of microorganisms at the genus level and clinical indices of children with pneumonia in the URT and LRT, respectively (Figure 6). The results showed that in URT, the abundance of *Streptococcus* was significantly correlated with the levels of white blood cells, lymphocytes, monocytes, basophils, and neutrophils. In LRT, the abundance of *Streptococcus* was significantly correlated with white blood cells, lymphocytes, monocytes, percentage of highly dispersed reticulocytes, basophils, immature reticulocyte index, eosinophils, absolute nucleated red blood cells, and basophils; however, the abundance of *Moraxella* was significantly correlated with absolute nucleated red blood cells, basophils, and activated fractional thromboplastin time; the abundance of Haemophilus was significantly correlated with thrombin time and procalcitonin; and the abundance of *mycoplasma* was significantly correlated with activated fractional thromboplastin time. In addition, we counted the respiratory parameters and the indexes of blood gas (Table S6) analysis of the subjects and performed a correlation analysis with the association between microbiome compositions. Notably, *Prevotella* in the upper airway was negatively correlated with pH and positively correlated with HCO_3_ levels and days of hospitalization.

## DISCUSSION

The respiratory microbiota balance is susceptible to external environmental influences (such as air pollution and antibiotic abuse), which can alter the microbiota in the respiratory tract and trigger the invasion of pathogenic bacteria, in turn causing a local immune imbalance in the respiratory tract, eventually leading to immune dysfunction.^1^, ^14^, ^15^, ^16^ The bacterial composition of the URT and LRTs belongs to the same species in healthy individuals,^17^, ^18^ with the abundance of microbiota being higher in the URT.^8^, ^19^ In the lower airways of healthy individuals, the most predominant phyla are the *Bacteroidetes* and the *Firmicutes*, and the most common genera include *Prevotella*, *Veronococcus*, and *Streptococcus*.^20^, ^21^ A study on young children reported that although the lung microbiota was distinct from that of the URT, it was dominated by species present in the URT, including *Moraxella*, *Haemophilus*, *Staphylococcus*, and *Streptococcus*.^22^ *Firmicutes* and *Proteobacteria* were the most dominant phyla in children with nonmycoplasma pneumonia.^13^ Our results showed that the four phyla with the highest abundance in the upper and lower airways of patients with pneumonia were identical: *Proteobacteria, Firmicutes, Actinobacteria*, and *Bacteroidetes*. This is comparable to another study in which the phyla *Firmicutes, Proteobacteria, and Actinobacteri*a were also the most abundant in the lungs of healthy individuals and those with asthma.^23^

In healthy individuals, the URT is higher than LRT in terms of bacterial abundance,^8^ and the respiratory microbiota in the LRT is transferred from the URT, meaning that no species differences exist between the URT and LRT. In our study, we examined pharyngeal swabs and BALF from pneumonia patients and found that the species richness in the LRT was significantly higher than that of the URT group, with a total of 21 families, including *Moraxellaceae, Corynebacteriaceae, and Rhodobacteraceae*. Montassier et al.^24^ found higher alpha diversity in oropharyngeal swabs than in BALF in patients with critical respiratory disease. In our study, we found a lack of microbiome difference in alpha diversity in URT versus LRT in the pharyngeal swabs and BALF of patients with pneumonia. Montassier's study included samples from critically ill patients with respiratory diseases, whereas we collected one‐to‐one samples from URT and LRTs of patients with pneumonia and did a cross‐sectional comparative analysis. The disparity in disease types could be a significant factor contributing to the differences in our findings. In addition, we enrolled patients with bacterial combined with viral infections, and the respiratory microbiota of this part may have been differently altered in the URT and LRT microbiota due to the presence of viruses. In addition, our sample size was limited, necessitating an expansion of our study population to obtain more reliable analyses, which is what we are going to address in our upcoming studies in the future.

For most bacterial pathogens, URT colonization was considered a prerequisite for causing pneumonia.^25^, ^26^ The conversion of these phyla to certain genera in the URT in patients with pneumonia can disrupt the LRT microbiota, leading to the proliferation of bacteria such as anaerobes and a massive invasion of foreign species, which ultimately results in ecological disorder in the host.^21^, ^27^ According to our results, *Pseudomonas* was significantly enriched in the LRT of children with pneumonia as well as in the BALF of lung transplant recipients compared to healthy subjects in a study by Dickson et al.^28^ *Pseudomonas* is an opportunistic pathogen that significantly contributes to morbidity and mortality, particularly in patients with cystic fibrosis and those with immunocompromised immune systems. It is a common cause of ventilator‐associated pneumonia,^29^ which poses a treatment challenge due to its high antibiotic resistance.^30^ Patients with pneumonia present different bacterial microbiota in the URT and LRT, so it is not feasible to speculate on changes in the LRT microbiota from changes in the URT microbiota only.

Pneumonia can be regarded as a disease that lies on a continuum of microbiota abundance, diversity, and composition with a corresponding set of immune states.^31^ As a complex system, even minor alterations can disrupt the state, leading to a cycle of positive and negative feedback loops, which subsequently leads to lung microbial dysbiosis.^32^ To further comprehend the relationship between the environmental microbiota and metabolite products and the impact of their metabolites on the immune response of the organism, metabolomics is now commonly employed to detect variations in metabolite structure and concentration in the environment, so as to reveal quantitative structural changes in the bacterial microbiota. Based on the 16S rDNA sequencing results, 50 metabolic pathways were identified, including d‐glutamine and d‐glutamate metabolism, lysine biosynthesis, and thiamine metabolism. One Ugandan study showed that the predominant bacterium was *Prevotellaceae* in LRT in HIV‐infected pneumonia patients, which resulted in significant enrichment of amino acid metabolites, monoacylglycerols associated with lipid metabolism, and inosine.^11^ Amino acid metabolism plays a key regulatory role in various immune responses.^33^, ^34^, ^35^ Dysregulation of amino acid metabolism holds promise as a biomarker for pneumonia.^36^ The metabolism of d‐glutamine by gut microbiota may affect cognitive function in patients with Alzheimer's disease.^37^ Gut microbiota plays an important role in lysine metabolism in humans.^38^ The association of thiamine, vitamin C, and corticosteroids was tested as an adjunctive therapy for septic shock, resulting in a significant decrease in mortality.^39^

Respiratory parameters and blood gas analysis indexes revealed intriguing correlations with the presence of *Prevotella* in the URT. Specifically, *Prevotella* was found to have an inverse relationship with pH levels and a positive correlation with HCO_3_ levels and days of hospitalization. *Prevotella* is a genus of anaerobic bacteria that are part of the phylum *Bacteroidetes*. These bacteria are commonly found in various environments, including the human body, where they inhabit the oral cavity, gastrointestinal tract, and female genital tract. Segal et al.^40^ reported that enrichment of *Prevotella* in BALF was associated with subclinical inflammation characterized by increased neutrophils and lymphocytes. Our results found that the upper respiratory microbiota may activate the lysine biosynthesis and thiamine metabolism metabolic pathways. Succinate is involved in lysine catabolism as an intermediate product of the TCA cycle; and thiamine (vitamin B1) is involved in succinate metabolism as a coenzyme. Rubic et al.^41^ reported that gut microbiota can produce considerable levels of succinate, succinate mainly produced by *Prevotella*. Therefore, we venture to speculate that *Prevotella* in the URT may be involved in lysine catabolism through the synthesis of succinic acid, which in turn contributes to thiamine metabolism, thus influencing the course of pneumonia in children.

Microbes from patients with pneumonia influence the metabolic processes of the body. Given the limitation of our sample size and the challenges to obtain alveolar lavage specimens of healthy volunteers, we made the decision not to include healthy volunteers into the analysis of this present study. Since the absence of samples from healthy children, we are unable to draw conclusions about their relationship to children's health. In addition, the use of antibiotics was not incorporated into the study for two primary reasons: First, samples were collected when the patients were admitted to the hospital or on the second day of admission, and second, the sample size was insufficient, which imposed significantly limitations on our analyses. Therefore, the new study that we are planning to carry out will include factors such as antibiotic use, time of administration, sample type, and time of patient process timing to do a more comprehensive analysis.

In summary, this study indicated that the upper and lower respiratory microbiota of children with pneumonia were significantly different, with the lower respiratory microbiota being significantly more abundant and diverse than the upper airways. Sixteen genera, including *Moraxella*, *Corynebacterium*, and *Propionibacterium*, were responsible for the significant differences between the URT and LRT. No significant differences were found between the bacterial and nonbacterial pneumonia groups. The predominant bacterium in the URT was *Streptococus* *infantis*, whereas the predominant groups in the lower tract are *Cyanobacteria, Alphaproteobacteria*, *Chloroplast*, *Pseudomonadales, Burkholderiales, Streptophyta, Moraxellaceae*, *Corynebacteriaceae*, *Moraxella* and *Corynebacterium*. The major metabolic pathways of the bacterium are d‐glutamine and d‐glutamate metabolism, lysine biosynthesis, thiamine metabolism, and 50 other metabolic pathways. *Streptococcus* spp. had a greater detrimental effect on routine blood indicators in both URT and LRT.

## CONFLICT OF INTEREST

None.

## Supporting information




**Table S1** The relative abundance of each pathway in each sample was obtained by mapping the functional gene abundance to the metabolic pathway (KEGG pathway, the KO level 3 taxonomic unit) to which it belongs.
**Table S2** Patient characteristics and outcomes.
**Table S3** Differential bacteria between URT and LRT in children with pneumonia.
**Table S4** Routine blood indicators.
**Table S5** Coagulation indicators.
**Table S6** Hematological indicators.

**Figure S1** The rarefaction curves plot of samples.
**Figure S2** Species accumulation analysis. Species accumulation plots showing the increase in OTUs detected with the addition of each patient sample. The left panel is the curve obtained using the OUTs of species. The right panel is the curve obtained using the OUTs of genus.
**Figure S3** The counts and percentage of the top 10 phyla between the URT and LRT.
