# Supplementary material for: Microbiota diversity and differences in the respiratory tract of children with pneumonia
Source: Pediatr Investig. 2025 Feb 14;9(3):262–74. doi: 10.1002/ped4.12466 (PMC12442450; doi:10.1002/ped4.12466)
Supplement: Supplementary file 2 — Figure S1 The rarefaction curves plot of samples. Figure S2 Species accumulation analysis. Species accumulation plots showing the increase in OTUs detected with the addition of each patient sample. The left panel is the curve obtained using the OUTs of species. The right panel is the curve obtained using the OUTs of genus. Figure S3 The counts and percentage of the top 10 phyla between the URT and LRT. [file PED4-9-262-s001.pdf]

Supplementary Material for

Microbiota diversity and differences in the respiratory tract of children with pneumonia

Dandan Ge, Lingyun Hou, Jintao Guo, Xuejing Lv, Yungang Yang

1 Sputum induction methods

The specific operation method of sputum induction by hypertonic saline nebulization: ①10 min before induction, let the patient inhale 400 ug of salbutamol, and inform the patient of the precautions for examination and the method of cooperation. ②After 10min, rinse the mouth with water and blow the nose. ③3% hypertonic saline nebulized inhalation for 15min, rinse the mouth and blow the nose, and then actively cough sputum into the culture dish. ④If the patient has no sputum or insufficient sputum, then switch to 4% hypertonic saline and continue nebulization for 8 min. ⑤If the patient has no sputum or insufficient sputum, then switch to 5% hypertonic saline and continue nebulization for 7 min and then terminate the induction procedure. ⑥ Nebulization was terminated if the patient coughed up enough qualified sputum specimen during the nebulization period or if the total duration of nebulization reached 30 min. Infants and young children were given a back tap by the examiner or a suction device was used to aspirate sputum.

Sputum cultures were performed in all patients with bacterial pneumonia, and an additional viral nucleic acid test or antibody test would be added in patients with non-bacterial pneumonia.

2 Supplementary Figures and Tables

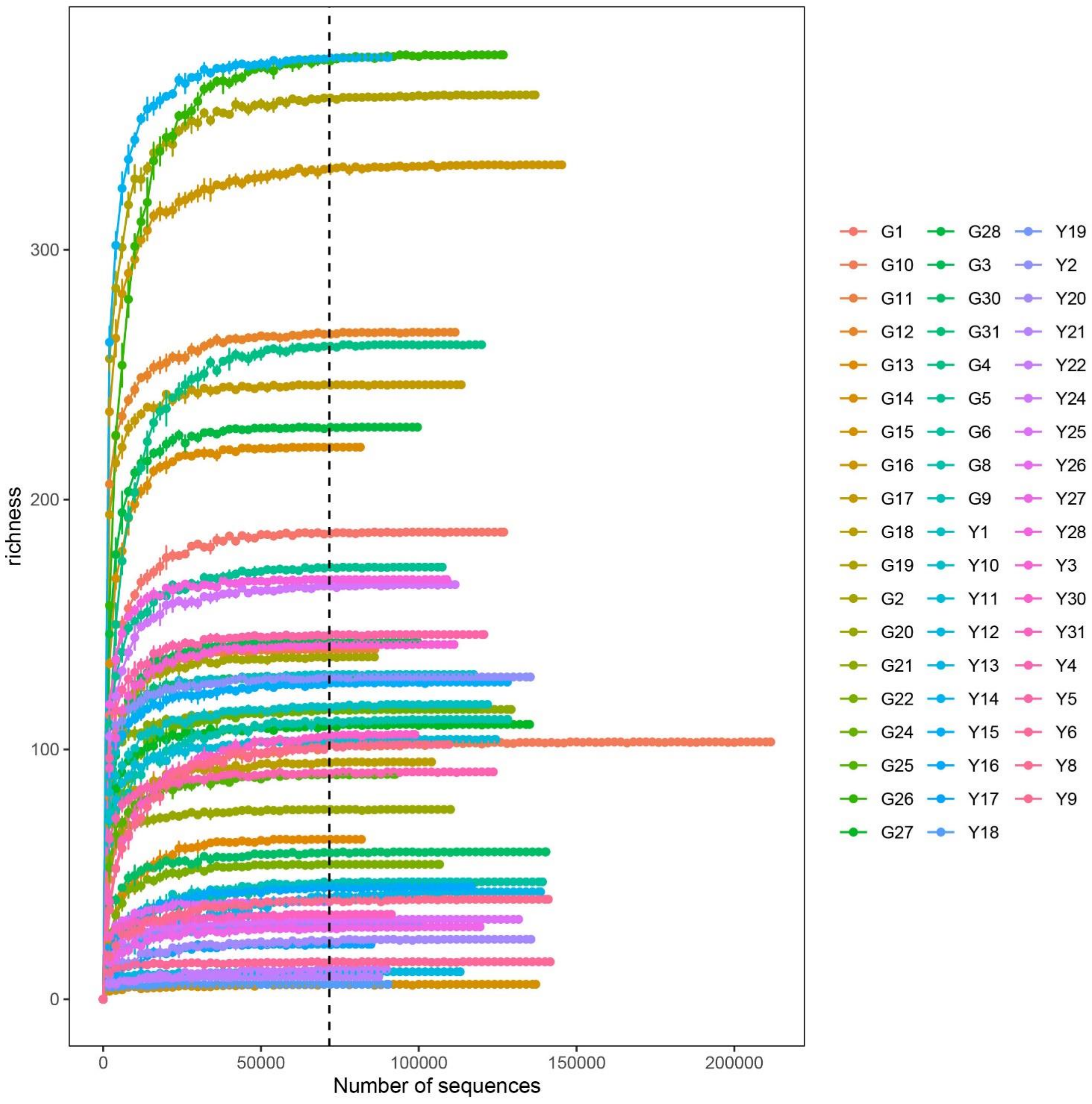

Supplementary Figure 1. The rarefaction curves plot of samples.

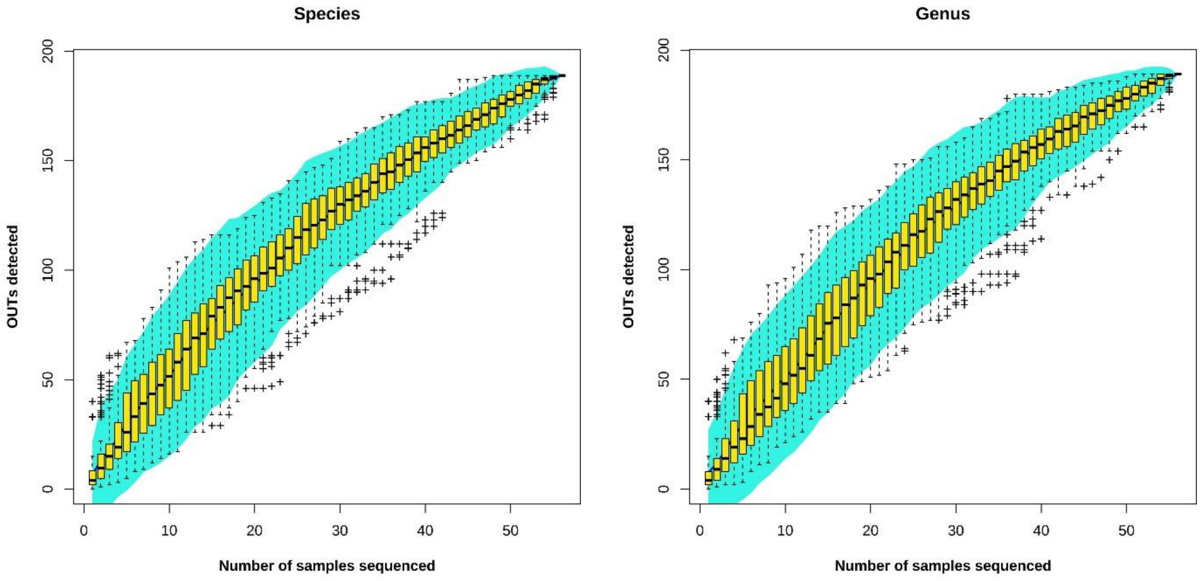

Supplementary Figure 2. Species accumulation analysis. Species accumulation plots showing the increase in OTUs detected with the addition of each patient sample. The left panel is the curve obtained using the OUTs of species. The right panel is the curve obtained using the OUTs of genus.

Taxonomy	Total(count)	Total	URT	LRT
p__Proteobacteria	2403465	40.91%	41.36%	40.47%
p__Firmicutes	1504290	25.61%	21.98%	29.23%
p__Actinobacteria	750440	12.77%	15.72%	9.83%
p__Bacteroidetes	543620	9.25%	9.67%	8.84%
p__Cyanobacteria	130742	2.23%	0.33%	4.12%
p__Tenericutes	121471	2.07%	2.83%	1.30%
p__Acidobacteria	85340	1.45%	1.27%	1.64%
p__TM7	56858	0.97%	1.66%	0.28%
p__Fusobacteria	50096	0.85%	1.08%	0.62%
Others	228127	3.88%	4.10%	3.67%

Supplementary Figure 3. The counts and percentage of the top 10 phyla between the URT and LRT.

Supplementary Tables

Supplementary Table 2. The relative abundance of each pathway in each sample was obtained by mapping the functional gene abundance to the metabolic pathway (KEGG pathway, the KO level 3 taxonomic unit) to which it belongs.

Supplementary Table 2. Patient characteristics and outcomes.

Supplementary Table 3. Differential bacteria between URT and LRT in children with pneumonia.

Supplementary Table 4. Routine blood indicators.

Supplementary Table 5. Coagulation indicators.

Supplementary Table 6. Hematological indicators.
